# Body composition is associated with tacrolimus pharmacokinetics in kidney transplant recipients

**DOI:** 10.1007/s00228-022-03323-0

**Published:** 2022-05-14

**Authors:** M. I. Francke, W. J. Visser, D. Severs, A. M. E. de Mik - van Egmond, D. A. Hesselink, B. C. M. De Winter

**Affiliations:** 1 Department of Internal Medicine, Division of Nephrology and Transplantation, Erasmus MC, University Medical Center Rotterdam, Room Rg-527, P.O. Box 2040, 3000 CA Rotterdam, The Netherlands; 2 Erasmus MC Transplant Institute, Rotterdam, The Netherlands; 3 Department of Hospital Pharmacy, Erasmus MC, University Medical Center Rotterdam, MC Rotterdam, The Netherlands; 4 Rotterdam Clinical Pharmacometrics Group, Rotterdam, The Netherlands; 5 Department of Internal Medicine, Division of Dietetics, Erasmus MC, University Medical Center Rotterdam, Rotterdam, The Netherlands

**Keywords:** Body composition, Kidney transplantation, Pharmacokinetics, Tacrolimus, Therapeutic drug monitoring

## Abstract

**Purpose:**

A population pharmacokinetic (popPK) model may be used to improve tacrolimus dosing and minimize under- and overexposure in kidney transplant recipients. It is unknown how body composition parameters relate to tacrolimus pharmacokinetics and which parameter correlates best with tacrolimus exposure. The aims of this study were to investigate which body composition parameter has the best association with the pharmacokinetics of tacrolimus and to describe this relationship in a popPK model.

**Methods:**

Body composition was assessed using bio-impedance spectroscopy (BIS). Pharmacokinetic analysis was performed using nonlinear mixed effects modeling (NONMEM). Lean tissue mass, adipose tissue mass, over-hydration, and phase angle were measured with BIS and then evaluated as covariates. The final popPK model was evaluated using goodness-of-fit plots, visual predictive checks, and a bootstrap analysis.

**Results:**

In 46 kidney transplant recipients, 284 tacrolimus concentrations were measured. The base model without body composition parameters included age, plasma albumin, plasma creatinine, *CYP3A4* and *CYP3A5* genotypes, and hematocrit as covariates. After full forward inclusion and backward elimination, only the effect of the phase angle on clearance (dOFV =  − 13.406; *p* < 0.01) was included in the final model. Phase angle was positively correlated with tacrolimus clearance. The inter-individual variability decreased from 41.7% in the base model to 34.2% in the final model. The model was successfully validated.

**Conclusion:**

The phase angle is the bio-impedance spectroscopic parameter that correlates best with tacrolimus pharmacokinetics. Incorporation of the phase angle in a popPK model can improve the prediction of an individual’s tacrolimus dose requirement after transplantation.

**Supplementary information:**

The online version contains supplementary material available at 10.1007/s00228-022-03323-0.

## Introduction

After kidney transplantation, patients are often administered a bodyweight-based tacrolimus starting dose, followed by therapeutic drug monitoring (TDM). However, tacrolimus has a narrow therapeutic range, and under- and overexposure are common in the early phase after transplantation using this dosing strategy [1–3]. Bodyweight correlates poorly with a patient’s tacrolimus dose requirement and overweight patients are at increased risk of overexposure following bodyweight-based dosing [4–8]. As tacrolimus is a lipophilic drug, its pharmacokinetics might correlate better with body composition parameters rather than bodyweight, such as hydration status or fat mass. However, how these are related to tacrolimus’ pharmacokinetics is not clear.

So far, few studies have investigated the relationship between an individual’s body composition and tacrolimus exposure after solid organ transplantation. These studies reported correlations between a patient’s fat mass and lean body mass and tacrolimus exposure and apparent volume of distribution, whereas no associations were found between body mass index (BMI) and pharmacokinetic parameters [9, 10].

Tacrolimus’ pharmacokinetics is affected by multiple factors, which can in part explain the variability in an individual’s tacrolimus dose requirement [11–15]. We and others showed that a pharmacokinetic model including such factors can improve tacrolimus dosing [13, 16, 17]. In a prospective clinical trial, a starting-dose algorithm effectively predicted kidney transplant recipients’ tacrolimus dose requirement in 58% of the patients [13, 16]. Residual variability in tacrolimus dose requirement might be partly explained by body composition parameters. Most previous studies estimated body composition parameters using bodyweight and height. Body composition parameters can be more reliably derived from bio-impedance spectroscopy (BIS) measurements, which is a simple and inexpensive bedside technique [18]. Moreover, BIS-derived phase angle (PA) can be calculated as the arc tangent of reactance over resistance. This measure relates to body cell mass, membrane integrity, and hydration status [19].

The aims of this study were to (1) investigate the relationship between body composition parameters estimated based on bodyweight and height and those measured using BIS; (2) investigate which body composition parameter has the best association with the pharmacokinetics of tacrolimus; and (3) describe this relationship in a population pharmacokinetic (popPK) model.

## Methods

### Patient population

This study is a post hoc analysis of a prospective study in which 46 adult kidney transplant recipients were included to evaluate the natural course of body composition after a kidney transplantation (not yet published). The study protocol was reviewed and approved by our medical ethical review board (MEC-2019–0723) and informed signed consent was obtained from all participants.

Patients were included in the present analysis if tacrolimus was part of their initial immunosuppressive regimen. After transplantation, patients were treated with oral twice-daily tacrolimus (Prograft®, Astellas Pharma, Leiden, The Netherlands), prednisolone, and mycophenolate mofetil.

### Study design and data collection

Body composition was assessed with the Body Composition Monitor (BCM, Fresenius Medical Care, Bad Homburg, Germany), which is based on BIS at 50 different frequencies ranging between 5 and 1000 kHz. The BCM has been validated against gold standard reference methods [20] and has the ability to differentiate between excess fluid and normally hydrated lean tissue mass [19]. The following parameters were recorded during each measurement: weight, height, lean tissue mass (LTM), adipose tissue mass (ATM), PA, and estimated over-hydration. Lean tissue index (LTI) and fat tissue index (FTI) were calculated respectively as LTM and ATM divided by height^2^ (kg/m^2^). BIS measurements were performed once for each patient using a standardized protocol and by experienced operators within 24 h before or three days after the transplantation.

Estimated body composition parameters were calculated using the following formulas:

- Body mass index (BMI)
  - BMI = (weight in kg)/(height in m)^2^
- Ideal body weight (IBW) [21]
  - Female: IBW = 49 + ((length in cm-152)*0.39)*1.7
  - Male: IBW = 52 + ((length in cm-152)*0.39)*1.9
- Lean tissue mass (LTM) [22]
  - Female: LTM = 1.07*weight in kg-148*(weight in kg/height in cm)^2^
  - Male: LTM = 1.1*weight in kg-128*(weight in kg/height in cm)^2^
- Lean tissue mass (LTM) for kidney transplant recipients [23]
  - Female: LTM = (10.2*weight in kg/(81.3 + weight in kg))*(1 + height in cm*0.052)*(1-age in years)*0.0007)
  - Male: LTM = (11.4*weight in kg/(81.3 + weight in kg))*(1 + height in cm*0.052)*(1-age in years)*0.0007)
- Adipose tissue mass (ATM)
  - weight in kg-LTM
- Body surface area (BSA) [24]
  - √ (height in cm*weight in kg/3600)

Data on a patient’s baseline characteristics (among which age, sex, height, weight), cytochrome P450 (*CYP*) *3A4* and *CYP3A5* genotype, and all pre-dose tacrolimus-, plasma albumin-, and plasma creatinine concentrations and hematocrit, measured in the first 3 weeks after kidney transplantation, were collected retrospectively from the electronic patient charts.

### Laboratory analysis

Tacrolimus concentrations were measured in whole-blood samples using a validated liquid chromatography-tandem mass spectrometry method (LC–MS/MS) in an ISO15189 certified laboratory. The imprecision of this method is < 10% with a bias < 15% over the validated range 1.0–35.0 ng/mL. Plasma albumin (bromocresol green method) and plasma creatinine were measured using the Cobas 8000 modular analyzer series (Roche Diagnostics, Almere, The Netherlands).

### Genotyping

If a patient’s *CYP3A4* and *CYP3A5* genotype was not already available, genotyping was performed in accordance with standard laboratory procedures in an ISO15189 certified laboratory. Samples were analyzed for the presence of the CYP3A4*1B, *2, *3, *6, *12, *17, *18, *20, and *22 and CYP3A5*2, *3, *6, *7, *8, and *9 polymorphisms using TaqMan Assay reagents for allelic discrimination (Applied Biosystems, San Diego, USA) with a 7900 Applied Biosystems thermal cycler.

### Study endpoints

The present study investigated (1) the relationship between estimated body composition parameters based on bodyweight and height and parameters measured using BIS and (2) which body composition parameter had the best association with the pharmacokinetics of tacrolimus and (3) if this relationship could be described in a popPK model.

### Statistical analysis

Statistical analyses were performed in R (version 4.0.1). Categorical variables were described as number of cases with a percentage. Non-normally distributed continuous variables were described as median with interquartile range (IQR). Correlations were calculated using Spearman’s correlation coefficient.

### Population pharmacokinetic modeling

Pharmacokinetic analysis was performed using nonlinear mixed effects modeling (NONMEM; version 7.4.4). PsN Pirana software (version 2.9.9) was used as an interface between NONMEM, R (version 4.0.1.), and Xpose (version 4.7.0.).

### Base model

A popPK model that we previously developed was used as base model for the present analysis [13]. This was a two-compartment model with first-order absorption, in which the values for lag‐time (t_lag_), absorption rate constant (k_a_), central volume of distribution (V_1_), peripheral volume of distribution (V_2_), clearance (CL), and inter-compartmental clearance (Q) were estimated. Since tacrolimus is administered orally, the bioavailability (F) could not be estimated, and therefore, F was fixed to 1 and all parameters are described as apparent values. Inter-individual variability (IIV) and inter-occasion variability (IOV) were modeled using an exponential model. An occasion was defined as the measurement of a tacrolimus pre-dose concentration. The model included a covariate effect of albumin, age, BSA, creatinine, hematocrit, *CYP3A4* genotype, and *CYP3A5* genotype on CL and a covariate effect of lean bodyweight on the central compartment V_1_. To be able to evaluate the effect of different body composition parameters on tacrolimus pharmacokinetics, BSA and lean bodyweight were excluded from the base model. Since all tacrolimus concentrations in the present study were measured using a LC–MS/MS method, additive and proportional errors for immunoassays were also removed from the base model. All other parameters were fixed to the final values of the model of Andrews et al. For each covariate, we evaluated whether re-estimating this fixed theta would improve the model (ΔOFV > 3.84) to check our assumption that the populations were similar.

### Covariate model

The present study investigated whether body composition parameters are correlated with the pharmacokinetics of tacrolimus. The following body composition parameters were evaluated as potential additional model covariates: BIS-derived ATM, FTI, LTM, LTI, over-hydration, PA, and estimated BMI, BSA, IBW, LTM, LTM for kidney transplant recipients, and ATM. Covariates were added using an exponential model.

A forward inclusion-backward elimination method was used for covariate modeling. All covariates were added to the base model in a univariate manner to evaluate their effect on CL/F, V_1_/F, and V_2_/F. Covariates were added to the full model if they significantly improved the base model (a decrease in objective function value (OFV) of > 3.84; *p* < 0.05). In the backward elimination step, covariates were excluded from the model if the decrease in OFV was below 6.64 (i.e., a significance level of *p* > 0.01). For the whole covariate analysis, all base model parameters were fixed, except for the ones that the covariate effect was estimated for either CL, V_1_, or V_2_, its variability (IIV and IOV), and the covariate that was added. This is because we had no available AUC of the patients in the present study and the sample size was small compared to the sample size which was used to build the base model (*n* = 337) [13].

### Model evaluation

To evaluate the effect of including a body composition parameter, the objective function, goodness-of-fit plots, parameter precision, shrinkage, visual predictive checks (VPCs), normalized prediction distribution errors (NPDE) analysis, and a bootstrap analysis were used. To compare the objective function between two models, a base model in which the fixed effect parameter (CL, V_1_ or V_2_) and its corresponding variability were estimated was compared to the same model including the effect of the potential covariate.

The model was internally validated by computing VPCs with 1000 simulations, stratified for the covariates that were evaluated for inclusion in the final model. The NPDE analysis was computed with 1000 simulations. Moreover, a bootstrap analysis was performed, with 1000 dataset samples.

### Model performance

To evaluate the model performance, we estimated the tacrolimus concentrations that patients would have had if they would have received an algorithm-based tacrolimus dose. Tacrolimus concentrations were estimated for different dosing strategies: (1) bodyweight-based dosing (0.2 mg/kg daily), (2) algorithm-based dosing according to the full model we previously developed [13], and (3) algorithm-based dosing according to the new model including body composition parameters. Tacrolimus concentrations were estimated by using the following formula:

$$Estimated\;tacrolimus\;concentration=\frac{Estimated\;tacrolimus\;dose\;\ast\;Observed\;tacrolimus\;concentration}{Administered\;tacrolimus\;dose}$$

For this analysis, the first steady state concentration of each patient was used (i.e., the concentration measured after 5 unaltered tacrolimus dosages). If the concentration was not measured in steady state, the patient was excluded from this analysis.

## Results

### Baseline characteristics

A total of 46 patients were included. Table 1 describes their baseline characteristics. The median age of the participants was 65 years (IQR 57.5–72.0) and 52% was male. The median BMI and BSA of the included patients were 28.0 kg/m^2^ (IQR 24.5–30.9) and 1.98 m^2^ (IQR 1.82–2.10), respectively. *CYP3A4* and *CYP3A5* genotypes were in Hardy–Weinberg equilibrium (*χ*^2^ = 0.17; *p* = 0.72; and *χ*^2^ = 0.12; *p* = 0.72, respectively). Five patients were carriers of a CYP3A4*22 allele, and 10 patients were considered CYP3A5 expressers (i.e., having at least one *1 allele). For 5 patients, the *CYP3A4* and *CYP3A5* genotype was unknown. For the analysis, we assumed that these patients had the genotype which is most common in our population (CYPA5*3/*3 plus CYP3A4*1/*1) [25, 26] (Supplementary Table S1 describes the baseline characteristics of the model-building population of the study by Andrews et al. [13]).

**Table 1:** Baseline characteristics

*Recipient characteristics*	*Study population (n* = *46)*
Gender	
Female/male	22 (48%)/24 (52%)
Age (years)	65.0 (IQR 57.5–72.0)
*CYP3A4* genotype	
*22 carrier/*22 non-carrier/missing	5 (10.9%)/36 (78.3%)/5 (10.9%)
*CYP3A5* genotype	
Expresser/non-expresser/missing	10 (21.7%)/31 (67.4%)/5 (10.9%)
Bodyweight (kg)	82.1 (IQR 71.6–92.2, range 46.0–119.5)
Height (cm)	170.0 (IQR 164.2–175.5, range 153.0–197)
BMI (kg/m^2^)	28.0 (IQR 24.5–30.9, range 18.9–39.4)
BSA (m^2^)	1.98 (IQR 1.82–2.10, range 1.41–2.56)
Estimated	
Ideal body weight (kg)	64.2 (IQR 57.1–68.3, range 49.7–85.4)
Lean body weight (kg)	56.7 (IQR 49.4–63.3, range 36.4–84.4)
Lean body weight KTR (kg)	52.2 (IQR 44.6–56.9), range 32.0–73.1)
Adipose tissue mass (kg)	23.4 (IQR 17.6–30.3, range 9.7–52.8)
BIS-derived	
Lean tissue mass (kg)	33.1 (IQR 27.9–42.27, range 19.7–73.4)
Lean tissue index (kg/m^2^)	11.9 (IQR 10.2–13.5, range 7.5–19.1)
Adipose tissue mass (kg)	44.4 (IQR 30.9–53.2, range 14.1–73.5)
Fat tissue index (kg/m^2^)	14.5 (IQR 11.0–18.02, range 2.0–27.7)
Phase angle (°)	4.8 (IQR 4.1–5.3, range 3.0–6.9)
Over-hydration (with 100 as reference)	102.3 (IQR 101.0–103.8, range 98.9–103.8)

Continuous variables are described as median (IQR, range). Categorical variables as number of cases (%)

*KTR* kidney transplant recipients

### Correlation between BIS and estimated body composition parameter

**Fig. 1:**
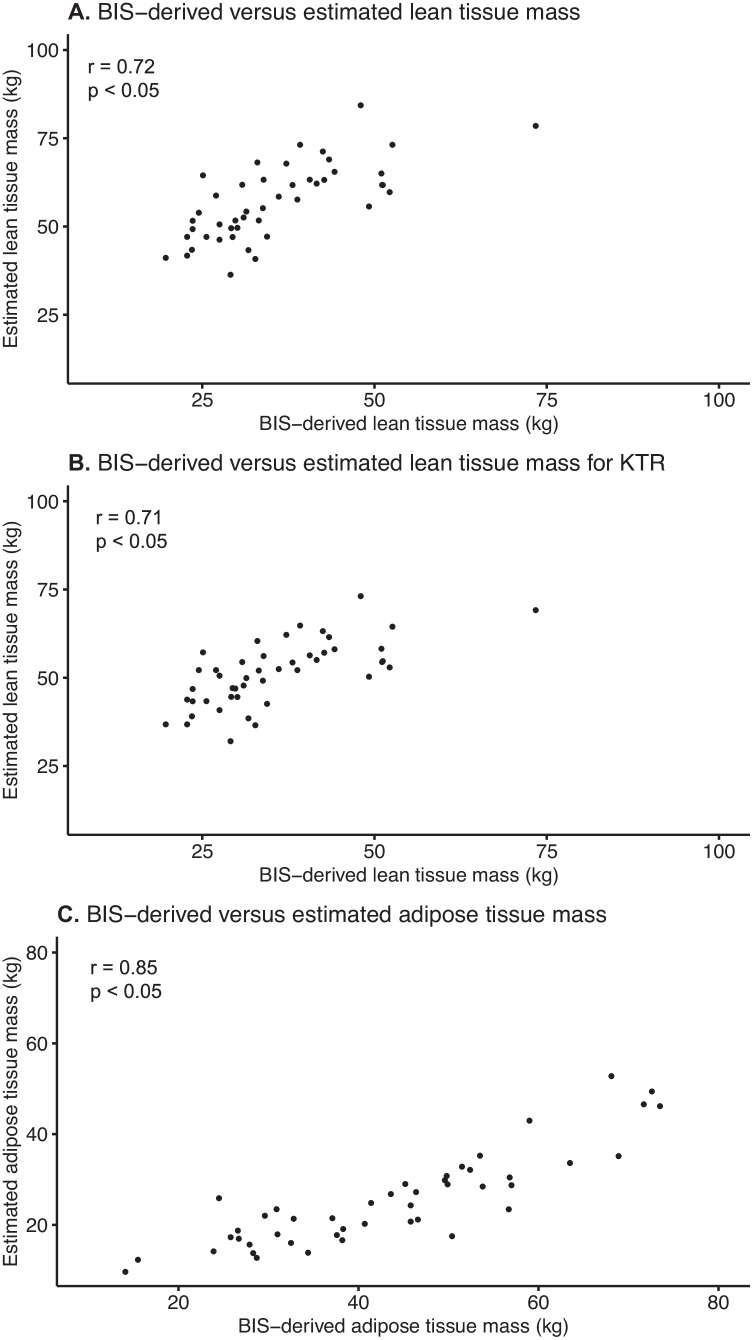
Correlations between the measured BIS-derived values and the estimated values of **A** lean tissue mass according to the formula by James et al. [22], **B** lean tissue mass for kidney transplant recipients according to the formula of Størset et al. [23], and **C** adipose tissue mass. BIS, bio-impedance spectroscopy; KTR, kidney transplant recipients; r, Spearman’s correlation coefficient

The correlation between the estimated LTM and LTM measured using BIS was moderate (Spearman’s *r* = 0.72; *p* < 0.05; Fig. 1A). Similarly, the correlation between the estimated LTM for kidney transplant recipients and LTM measured using BIS was moderate (Spearman’s *r* = 0.71; *p* < 0.05; Fig. 1B). The correlation between the estimated ATM and ATM measured using BIS was better (Spearman’s *r* = 0.85; *p* < 0.05; Fig. 1C). However, the formula appears to systemically find a lower ATM than BIS.

### Body composition and tacrolimus clearance

In the 46 included patients, 284 tacrolimus concentrations were measured in the first 3 weeks after kidney transplantation. Six samples of three patients were above the limit of quantification (35 ng/mL), and their values were extrapolated from the raw LC–MS/MS data using the calibration curve of the validated concentration range. Re-estimating the covariates (age, albumin, creatinine, hematocrit, *CYP3A4* genotype, and *CYP3A5* genotype) did not significantly improve the model fit for any of the covariates (ΔOFV < 3.84).

### Covariate analysis

**Fig. 2:**
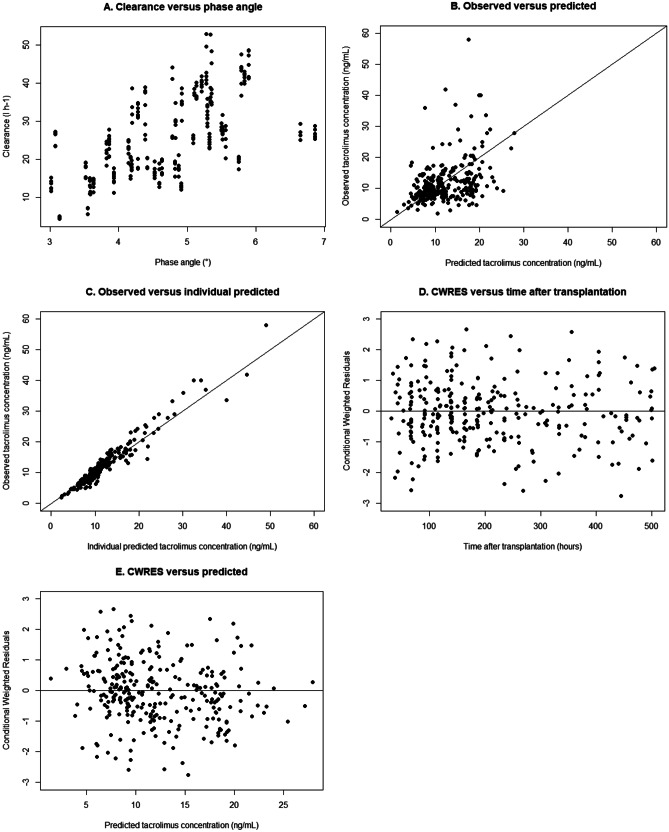
Goodness of fit plots of the final model. **A** The relationship between the phase angle and the clearance of tacrolimus. **B** Observed tacrolimus concentrations versus predicted tacrolimus concentrations. **C** Observed tacrolimus concentrations versus the individual predicted tacrolimus concentrations. **D** The conditional weighted residuals over the time after transplantation. **E** The conditional weighted residuals over the predicted tacrolimus concentrations. CWRES, conditional weighted residuals

For the covariate analysis, the base model (described in Table 2) was used as reference model. In the covariate analysis (Table 3), body composition parameters were separately added to the base model, and included if they significantly improved the base model (a decrease in OFV of > 3.84; *p* < 0.05). A significant effect on CL/F was observed for LTM (dOFV =  − 6.247, theta = 0.577), LTI (dOFV =  − 5.448, theta = 0.691), and PA (dOFV =  − 13.406, theta = 1.22, Fig. 2E). None of the covariates significantly correlated with V_1_/F or V_2_/F (with a reference model in which V_1_ and V_2_ were estimated instead of fixed). After full forward inclusion, covariates were excluded from the model if the decrease in OFV was below 6.64 (i.e., *p* > 0.01). After full forward inclusion and backward elimination, only the effect of the PA on CL/F was included in the final model (Table 3; Supplementary Data S1). The tacrolimus CL/F is estimated by the following equation based on the final model:

**Table 2:** Model parameter estimates

*Parameter*	*Final model Andrews *et al.	*Base model*	*Final model*	*Bootstrap final model* *Median (95% range)*
t^lag^ (h)	0.38	0.38 (f)	0.38 (f)	0.38 (f)
K_a_ (l h^−1^)	3.58	3.58 (f)	3.58 (f)	3.58 (f)
CL/F (l h^−1^)	23.0	24.9	26.1	26.1 (23.5–28.9)
V_1_/F(l)	692	692 (f)	692 (f)	692 (f)
Q/F (l h^−1^)	11.6	11.6 (f)	11.6 (f)	11.6 (f)
V_2_/F (l)	5340	5340 (f)	5340 (f)	5340 (f)
Covariate effect on CL				
CYP3A5*1	1.63	1.63 (f)	1.63 (f)	1.63 (f)
CYP3A4*22	0.80	0.8 (f)	0.8 (f)	0.8 (f)
Hematocrit (ll^−1^)	− 0.76	− 0.76 (f)	− 0.76 (f)	− 0.76 (f)
Creatinine (µmol/l)	− 0.14	− 0.14 (f)	− 0.14 (f)	− 0.14 (f)
Albumin (g l^−1^)	0.43	0.43 (f)	0.43 (f)	0.43 (f)
Age (years)	− 0.43	− 0.43 (f)	− 0.43 (f)	− 0.43 (f)
BSA (m^2^)	0.88	-	-	-
Phase angle (°)	-	-	1.22	1.18 (0.45–2.15)
Covariate effect on V_1_				
Lean tissue mass (kg)	1.52	-	-	-
IIV (%)				
CL/F	38.6	41.7 [12.4%]	34.2 [15.2%]	31.5 (14.6–45.8)
V_1_/F	49.2	49.2 (f)	49.2 (f)	49.2 (f)
V_2_/F	53.0	53.0 (f)	53.0 (f)	53.0 (f)
Q/F	78.7	78.7 (f)	78.7 (f)	78.7 (f)
IOV (%)				
CL/F	13.6	11.4 [63.8%]	11.4 [63.7%]	11.7 (2.7–18.8)
Residual variability				
Proportional (%)				
Immunoassay	17.7	-	-	-
LC–MS/MS	24.5	16.3	16.3	16.2 (12.8–18.8)
Additive				
Immunoassay	1.02	-	-	-

(f) indicates the fixed parameters

[shrinkage]

*CL* clearance, *CYP* cytochrome P450, *F* bioavailability of oral tacrolimus, *IIV* inter-individual variability, *IOV* inter-occasion variability, *K*_*a*_ absorption rate constant, *LC–MS/MS* liquid chromatography-tandem mass spectrometry, *Q* inter-compartmental clearance of tacrolimus, ^*t*^*lag* lag time, *V*_*1*_ central compartment for tacrolimus, *V*_*2*_ peripheral compartment for tacrolimus

**Table 3:** Estimates forward inclusion model building

*Covariate tested*	*Theta*	*dOFV*
*Effect on V1*^a^		
Body mass index	− 0.18	− 0.044
Body surface area	− 0.519	− 0.424
BIS-derived lean tissue mass	0.0763	− 0.031
BIS-derived lean tissue index	0.489	− 0.718
Estimated lean tissue mass	− 0.312	− 0.366
Estimated lean tissue mass KTR	− 0.107	− 0.159
BIS-derived adipose tissue mass	− 0.284	− 0.818
BIS-derived fat tissue index	− 0.278	− 0.49
Estimated adipose tissue mass	− 0.091	− 0.044
Ideal body weight	− 0.576	− 0.618
Phase angle	0.211	− 0.037
Overhydration + 100	3.3	− 0.401
Effect on V2^b^		
Body mass index	1.77	− 1.318
Body surface area	1.95	− 1.675
BIS-derived lean tissue mass	1.23	− 1.736
BIS-derived lean tissue index	0.898	− 0.744
Estimated lean tissue mass	1.15	− 1.352
Estimated lean tissue mass KTR	0.672	− 0.292
BIS-derived adipose tissue mass	0.415	− 0.642
BIS-derived fat tissue index	0.236	− 0.34
Estimated adipose tissue mass	0.637	− 0.968
Ideal body weight	1.33	− 0.944
Phase angle	0.804	− 0.6
Overhydration + 100	− 1.44	− 0.017
Effect on CL^c^		
Body mass index	− 0.0874	− 0.049
Body surface area	0.747	− 1.72
**BIS-derived lean tissue mass**	**0.577**	** − 6.247**
**BIS-derived lean tissue index**	**0.691**	** − 5.448**
Estimated lean tissue mass	0.519	− 2.013
Estimated lean tissue mass KTR	0.102	− 0.416
BIS-derived adipose tissue mass	− 0.0282	− 0.024
BIS-derived fat tissue index	− 0.104	− 0.501
Estimated adipose tissue mass	0.033	− 0.033
Ideal body weight	0.678	− 1.859
**Phase angle**	**1.22**	** − 13.406**
Overhydration + 100	− 5.4	− 2.838

*dOFV* difference in objective function value compared to the reference model, *CL* clearance, *KTR* kidney transplant recipients, *V1* central volume of distribution, *V2* peripheral volume of distribution

Covariates were added following the following formulas

^a^V1 = V1population * (Covariate/Median)^Theta^

^b^V2 = V2population * (Covariate/Median)^Theta^

^c^CL = CLpopulation * (Covariate/Median)^Theta^

$$\begin{aligned}CL/F=26.1\;&\ast\;\left[\left(1.0,if\,CYP3A5\ast3/\ast3\right)\;or\;\left(1.631,\;if\;CYP3A5\ast1/\ast3\;or\;CYP3A5\ast1/\ast1\right)\right]\;\\& \ast\;\left[\left(1.0,if\,CYP3A4\;\ast\;1\;or\;unknown\right)\;or\;\left(0.8,\;if\;CYP3A4\;\ast\;22\right)\right]\;\ast\;\left(\frac{Age}{56}\right)^{-0.43}\;\\&\ast\;\left(\frac{Albumin}{42}\right)^{0.43}\;\ast\;\left(\frac{Creatinine}{135}\right)^{-0.14}\;\ast\;\left(\frac{Hematocrit}{0.34}\right)^{-0.76}\;\ast\;\left(\frac{PhaseAngle}{4.8}\right)^{1.22}\end{aligned}$$

### Model evaluation

The final model was evaluated using goodness-of-fit plots, parameter precision, shrinkage, visual predictive checks (VPCs), and a bootstrap analysis.

Figure 2 shows the goodness-of-fit plots. The (individual) model predictions were evenly distributed around the line of unity, and the conditional weighted residuals did not show a trend over time or over the predicted tacrolimus concentrations.

The IIV in the clearance decreased from 41.7% in the base model to 34.2% in the final model. This corresponds with a decrease in IIV of 32.8%. This means that the final model, including the PA, can further explain variability between patients in the tacrolimus pharmacokinetics. Supplementary Figure S1 shows the eta on clearance versus PA before and after the inclusion of PA in the model. The shrinkage for the IIV was 15.2%, and the shrinkage for the IOV was 63.7%. We accepted the high value for shrinkage for IOV because the estimated value was similar to the original model.

**Fig. 3:**
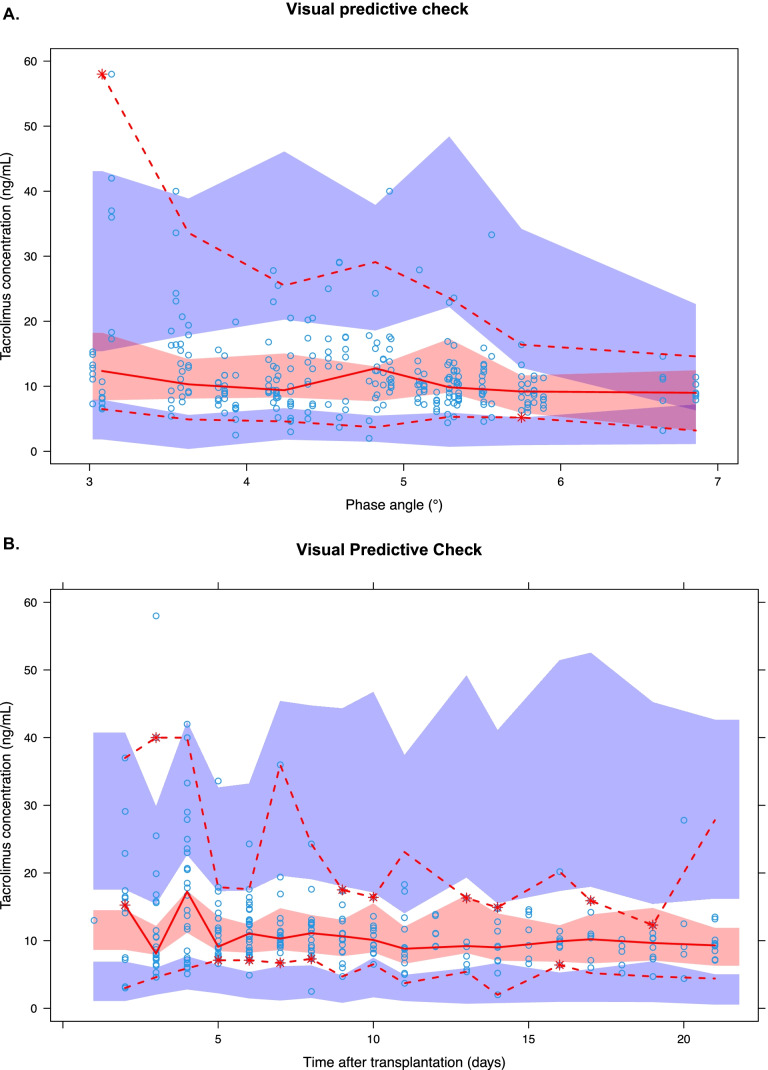
Visual predictive check showing how well the mean of the observed tacrolimus concentrations (red line) falls within the predicted mean tacrolimus concentration (red area; 95% confidence interval) and how well the variability of the observed tacrolimus concentration (red-dotted line) falls within the predicted variability of the tacrolimus concentration (blue area; 95% confidence interval) over **A** the phase angle, and **B** the time after transplantation

The VPCs (Fig. 3) show that the median observed tacrolimus concentrations fall within the 95% confidence interval of the simulated median tacrolimus concentration. Moreover, the observed variation fell within the 95% confidence interval of the simulated variation. Only for a low PA, the observed variation was outside the 95% confidence interval of the simulated variation, which was caused by one extreme value.

**Fig. 4:**
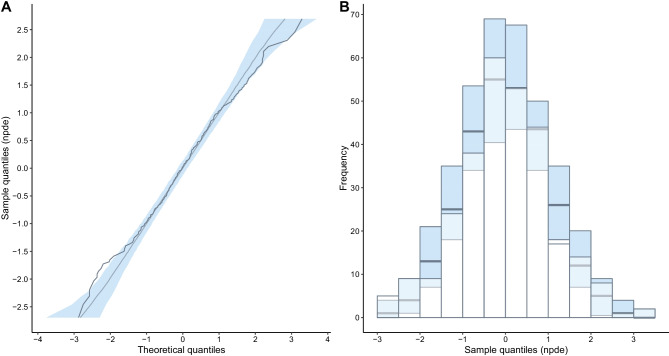
QQ plot **A** and histogram **B** showing the normality of the normalized prediction distribution errors (NPDEs) distribution for the final model

In the NPDE QQ plot (Fig. 4A), the data follows the theoretical line and largely fits within the confidence interval. The NPDE histogram (Fig. 4B) overlaps with the theoretical normal distribution.

In the bootstrap analysis (Table 2), the medians of the estimated parameters were similar to the estimates in the final model and were within the 90-percentile range.

### Model performance

**Fig. 5:**
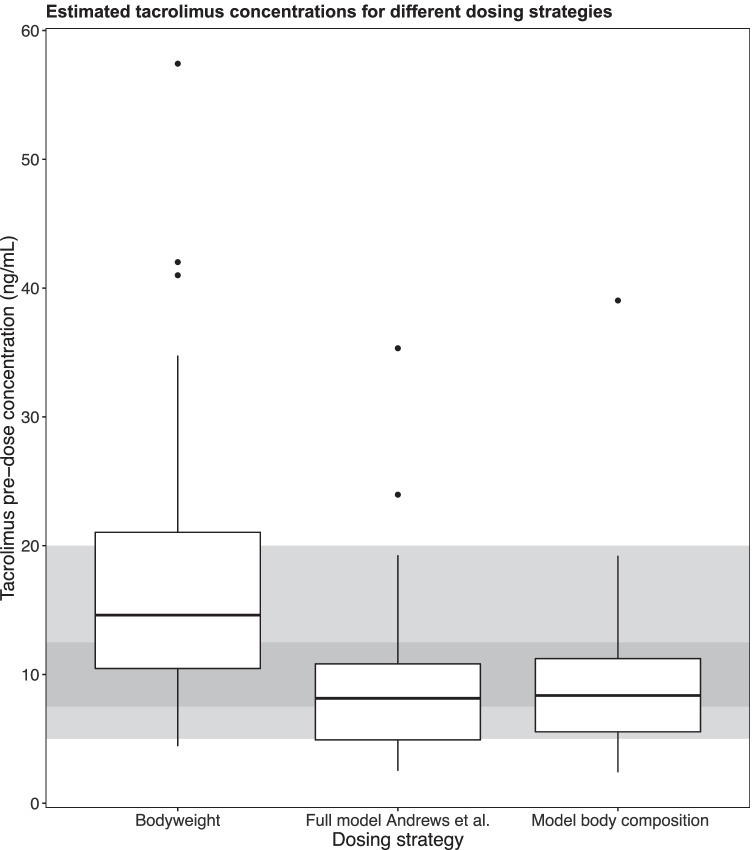
Boxplot of the estimated tacrolimus concentrations following different dosing strategies. The dark-gray area represents the tacrolimus target range (7.5–12.5 ng/mL), the the light-gray areas represent the areas of moderate underexposure (5.0–7.5 ng/mL) and overexposure (12.5–20.0 ng/mL)

Of the 41 patients included in this analysis, 9 (22%) were estimated to have had a therapeutic tacrolimus concentration if their dose would have been based on bodyweight alone. By using our previously published dosing algorithm [13], 16 patients (39%) were estimated to have had a therapeutic tacrolimus concentration, and by using the new dosing algorithm including body composition parameters, 15 patients (37%; Table 4; Fig. 5). The algorithms especially seemed to reduce tacrolimus overexposure. With a bodyweight-based dose, extreme underexposure (< 5.0 ng/mL) was estimated to occur in 1 patient (2%), and extreme overexposure (> 20.0 ng/mL) was estimated to occur in 12 patients (29%). With an algorithm-based dose according to our previously published model [13] and according to the new model, extreme underexposure was estimated to occur in 11 (27%), and 7 patients (17%), respectively, and extreme overexposure was estimated to occur in 2 (5%) and 1 (2%) patients, respectively.

**Table 4:** Estimated tacrolimus exposure

	*Extreme underexposure* < *5.0 ng/l* *(n* = *41)*	*Underexposure* < *7.5 ng/l* *(n* = *41)*	*On target* *7.5–12.5 ng/l* *(n* = *41)*	*Overexposure* > *12.5 ng/l* *(n* = *41)*	*Extreme overexposure* > *20.0 ng/l* *(n* = *41)*
Bodyweight	1 (2%)	5 (12%)	9 (22%)	27 (66%)	12 (29%)
Full model Andrews et al. [13]	11 (27%)	19 (46%)	16 (39%)	6 (15%)	2 (5%)
Model body composition	7 (17%)	18 (44%)	15 (37%)	8 (20%)	1 (2%)

## Discussion

This study demonstrates that a patient’s body composition is associated with the pharmacokinetics of tacrolimus and can potentially improve the prediction of an individual’s tacrolimus dose requirement after kidney transplantation. The BIS-derived PA most strongly related to tacrolimus pharmacokinetics, and thus a patient’s dose requirement. To the best of our knowledge, this is the first study that demonstrates this relationship.

After kidney transplantation, patients are usually administered a bodyweight-based tacrolimus starting dose, followed by individual dose titration based on whole-blood concentrations. However, this approach may not be appropriate since body weight and BMI are poor predictors of tacrolimus clearance [4, 10]. A patient’s body composition may correlate better with an individual’s tacrolimus dose requirement. Within the field of oncology, the relation between body composition and medication dosage and toxicity was demonstrated previously [27]. Muscle mass was an especially important body composition parameter in terms of required chemotherapy dose and toxicity [28–30]. Muscle mass has also been identified as a predictor of tacrolimus clearance [31, 32].

This is the first study that shows the relationship between PA and tacrolimus pharmacokinetics after kidney transplantation. Our final model demonstrates a positive effect of the PA on CL. With this final model, the IPV in CL decreases compared to the base model. This means that including the PA in the model can explain a part of the variability between patients in the pharmacokinetics of tacrolimus, and thus in the patients’ dose requirement. Although the differences in estimated target attainment between our previous model of and the model including PA are small, extreme under- and overexposure were estimated to occur less frequently by including PA in the model. A potential explanation for the relationship between PA and CL may come from what PA reflects. It is the ratio of the arc tangent of reactance to resistance and is related to important cellular characteristics, including membrane capacitance, integrity, permeability, overall size, hydration, and body cell mass, and the ratio between extracellular and intracellular fluid [19]. PA was shown to be a prognostic indicator of disease and/or nutrition risk in hemodialysis patients [33] and PA also appears to be a predictor of mortality in kidney transplant recipients [34]. Moreover, over-hydration is common in patients with kidney failure and the PA and over-hydration are negatively correlated [35, 36]. Patients with a better kidney function are in general less over-hydrated, and kidney function has in turn been associated with higher tacrolimus clearance [13].

A possible explanation for the effect of PA in the final model and especially on CL/F could be that there is a correlation between PA and the activity of CYP3A. Tacrolimus is metabolized by CYP3A, so higher activity of these enzymes leads to higher clearance. Zarezadeh et al. performed a systematic review about the effect of obesity, diet, and nutritional status on cytochrome P450 [37]. They concluded that obesity and overweight decrease the activity of CYP3A. Studies in malnourished patients show that drug metabolism can be limited and toxicity is influenced by nutritional status [38–41]. Furthermore, both CYP3A activity and PA are negatively correlated with inflammation [42, 43]. The correlation between PA and CYP3A activity has not been studied. We hypothesize that because of the relationship between nutritional status and CYP3A activity and drug metabolism, and the relationship between nutritional status and PA, there could be a correlation between PA and CYP3A activity and drug metabolism.

Another explanation for the relationship between PA and CL/F is a patient’s muscle mass, which is positively correlated with both PA and whole blood concentrations of tacrolimus [10, 44]. We observed a significant effect on clearance for LTM, LTI, and PA. Although muscle mass seems to have an important role in the pharmacokinetics of tacrolimus and the apparent volume of distribution, we found that including LTM in addition to the PA did not improve the model any further both in terms of volume of distribution (V_1_/F and V_2_/F) and CL/F. This indicates that PA and LTM are correlated. This is in line with the results of a study by Kosoku et al. who observed an association between sarcopenia, thus a low muscle mass, and PA in kidney transplant recipients [44].

The link between body composition, muscle mass, and the pharmacokinetics of tacrolimus was also reported by Han et al. [10]. They concluded that tacrolimus dosing based on body composition may provide adequate dosage leading to favorable long-term outcomes. They observed significantly higher whole blood tacrolimus concentrations in patients with a higher muscle mass compared to patients with a lower muscle mass. Sawamoto et al. [8] found that the steady-state pre-dose concentration of tacrolimus dose in obese patients was well maintained by a relatively low dose compared with that in normal-weight and lean patients. This result is supported by our previous work [4]. The possible explanation for these observations are multifactorial and may partly be related to the amount of muscle mass and the ratio between fat-mass and fat-free mass. Body weight does not reflect the amount of muscle mass nor the ratio between fat-mass and fat-free mass.

Potentially, when the ratio between fat mass and fat-free mass varies more, the likelihood of incorrect dosing will be most pronounced. The ratio is most skewed in sarcopenic obesity, where low muscle mass occurs in combination with high fat mass. In chemotherapy, a series of studies demonstrated this phenomenon and reported an association of dose-limiting toxicity with sarcopenic obesity in different treatment settings, potentially based on greater exposure during cancer treatment [29, 45–48]. Low muscle mass and the loss of muscle mass is a common finding in patients with kidney failure [49, 50] and dialysis patients [51, 52]. Sarcopenic obesity is common in kidney patients in all disease stages [50]. Moreover, after a kidney transplant, the body composition also changes unfavorably [53, 54]. Moreay et al. [53] found a stable LTM and an increase in fat mass. Habedank et al. [54] found that after kidney transplantation, adipose tissue increases and LTM decreases. These changes in body composition after kidney transplantation may also affect dose requirements.

Since tacrolimus is a highly lipophilic drug and the distribution of tacrolimus is predominantly in fat-rich organs [55]; the expectation would be that patients with a higher fat mass will have a higher apparent volume of distribution. Chen et al. [9] performed a study on the impact of body composition on the pharmacokinetics of tacrolimus in liver transplant recipients. They found in 80 liver transplant recipients that patients with a high body fat percentage (> 30%) had a lower apparent volume of distribution compared to patients with a low body fat percentage (< 30%). This counterintuitive finding was also found by Miyamoto et al. [56]. We found that none of the covariates significantly correlated with volume of distribution. Potential explanations for this result could be the difference in population, sample size, or the difference between estimated body fat percentage and BIS-derived ATM.

There are several methods of assessing muscle mass and ATM, including estimations based on body weight and height, and BIS, which has demonstrated good precision compared with gold standard methods [20]. We found that the estimated values of LTM and ATM are moderately correlated with those measured using BIS. Also, the correlation between the estimated LTM for kidney transplant recipients and LTM measured using BIS was moderate. Moreover, the estimated LTM were not significantly associated with tacrolimus clearance, where the BIS-derived LTM was. Consequently, these cannot be used interchangeably. BIS is relatively inexpensive, noninvasive, easy to perform, and validated for patients with kidney failure, and can therefore easily be implemented in clinical practice [19, 20]. Moreover, an additional advantage of measuring body composition with BIS is the determination of PA.

This study has some limitations. First, the sample size was relatively small compared to the previous dataset which was used to build the base model (*n* = 337). The sample size could also be the explanation for the high shrinkage for the IOV (63.7%). This indicates that the model cannot estimate the inter-occasion variability very well. However, as the model estimate of the IOV is similar to that of our previous model, [13] we assume that the estimate is reasonable. Because of the relatively small sample size, we may need more data to make a more precise and valid conclusion of the effect size of the body composition parameters, before including these variables in a model that can be used in clinical practice. Second, we only measured tacrolimus pre-dose concentrations and no area under the curve (AUC). This makes it more difficult to estimate the real tacrolimus exposure in the patients and their volume of distribution based on the data. As a consequence, we needed to fix some parameters based on a previously developed model, which used data of a larger population of kidney transplant recipients and included AUC measurements [13]. We think that this was reasonable, since the population for the previous model was similar to the population in this study considering the patients’ age, gender, height, body weight, BSA, and percentage of *CYP3A* genotype (Supplementary Table S1).

In summary, this study demonstrates that a patient’s body composition is associated with the pharmacokinetics of tacrolimus and can potentially improve the prediction of an individual’s tacrolimus dose requirement after transplantation. To confirm the hypothesis, a prospective study is needed. The BIS-derived PA is most strongly related to tacrolimus pharmacokinetics and should therefore be evaluated as covariate in future popPK models, alongside AUC measurements of tacrolimus concentration.

## Supplementary information

Below is the link to the electronic supplementary material.

Supplementary file1 (DOCX 49 KB)

## Data Availability

The data that support the findings of this study are available from the corresponding author upon reasonable request.
