# Supplementary material for: Body composition is associated with tacrolimus pharmacokinetics in kidney transplant recipients
Source: Eur J Clin Pharmacol. 2022 May 14;78(8):1273–87. doi: 10.1007/s00228-022-03323-0 (PMC9283366; doi:10.1007/s00228-022-03323-0)
Supplement: Supplementary file 1 — Supplementary file1 (DOCX 49 KB) [file 228_2022_3323_MOESM1_ESM.docx]

**SUPPLEMENTARY DATA**

**Supplementary Table S1.** Baseline characteristics of the model-building cohort of the present study and the study by Andrews et al.

**Supplementary Figure S1.** The ETA on clearance versus the phase angle (A) before and (B) after inclusion of phase angle in the model

**Supplementary Data S1.** NONMEM code

| **Table S1.** Baseline characteristics model-building cohorts | | |
| --- | --- | --- |
| *Recipient characteristics* | *Study population (n = 46)* | *Study population Andrews et al (n = 337)* |
| Gender  Female / Male | 22 (48%) / 24 (52%) | 133 (39.5%) / 204 (60.5%) |
| Age (years) | 65.0 (IQR 57.5 – 72.0) | 55.7 (IQR 43.3 – 64.0) |
| *CYP3A4* genotype  **22* carrier / *22 non-carrier /missing | 5 (10.9%) / 36 (78.3%) / 5 (10.9%) | 31 (9.2%) / 296 (87.8%) / 0 |
| *CYP3A5* genotype  Expresser / non-expresser/missing | 10 (21.7%) / 31 (67.4%) / 5 (10.9%) | 86 (25.5%) / 251 (74.5%) / 0 |
| Bodyweight (kg) | 82.1 (IQR 71.6 – 92.2, range 46.0 – 119.5) | 77.3 (IQR 68.0 – 88.0, range 37.6-132.0) |
| Height (cm) | 170.0 (IQR 164.2 – 175.5, range 153.0 - 197) | 173 (IQR165 – 182, range 141 – 203) |
| BMI (kg/m^2^) | 28.0 (IQR 24.5 – 30.9, range 18.9 – 39.4) | 25.7 (IQR 23.1 – 29.1, range 15.6 – 42.2) |
| BSA (m^2^) | 1.98 (IQR 1.82 – 2.10, range 1.41 – 2.56) | 1.93 (IQR 1.78 – 2.09, range 1.24 – 2.66) |
| Estimated |  |  |
| Ideal body weight (kg) | 64.2 (IQR 57.1 – 68.3, range 49.7 – 85.4) | 62.9 (IQR 57.6 – 68.9, range 41.7 – 82.8) |
| Lean body weight (kg) | 56.7 (IQR 49.4 – 63.3, range 36.4 – 84.4) | 57.5 (IQR 48.6 – 64.4, range 30.7 – 85.3) |
| Lean body weight KTR (kg) | 52.2 (IQR 44.6 – 56.9), range 32.0 – 73.1) | 52.6 (IQR 44.3 – 58.0, range 27.5 – 74.6) |
| Adipose tissue mass (kg) | 23.4 (IQR 17.6 – 30.3, range 9.7 – 52.8) | 19.3 (IQR 15.2 – 26.2, range 6.5 – 57.7) |
| BIS-derived |  |  |
| Lean tissue mass (kg) | 33.1 (IQR 27.9 – 42.27, range 19.7 – 73.4) | - |
| Lean tissue index (kg/m^2^) | 11.9 (IQR 10.2 – 13.5, range 7.5 – 19.1) | - |
| Adipose tissue mass (kg) | 44.4 (IQR 30.9 – 53.2, range 14.1 – 73.5) | - |
| Fat tissue index (kg/m^2^) | 14.5 (IQR 11.0 – 18.02, range 2.0 – 27.7) | - |
| Phase Angle (˚) | 4.8 (IQR 4.1 – 5.3, range 3.0 – 6.9) | - |
| Over-hydration (with 100 as reference) | 102.3 (IQR 101.0 – 103.8, range 98.9 – 103.8) | - |
| Continuous variables are described as median (IQR, range). Categorical variables as number of cases (%).  KTR, kidney transplant recipients | | |
|  | |  |


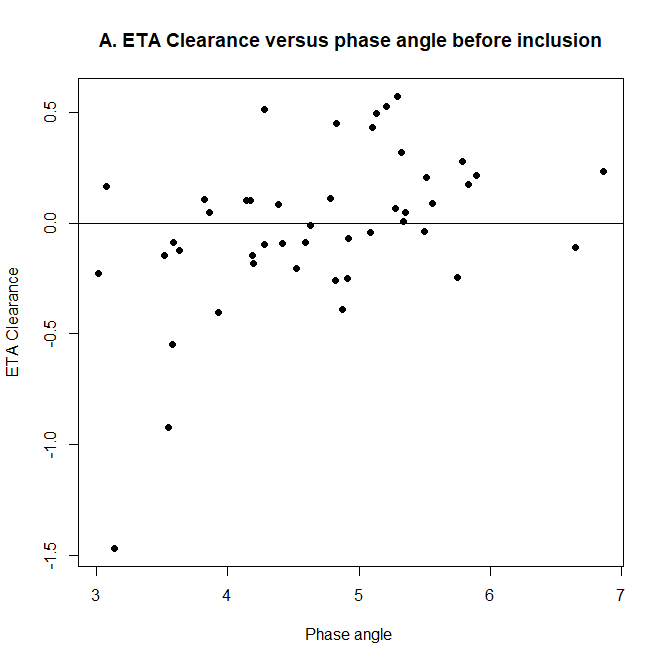

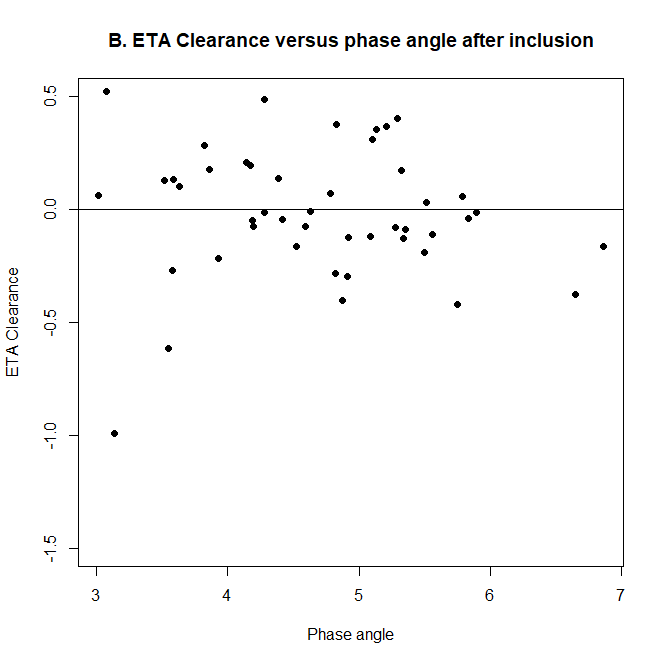


**Supplementary Figure S1.** The eta on clearance versus the phase angle (A) before and (B) after inclusion of phase angle in the model

**Supplementary Data S1.** NONMEM code

----------------------------------- INPUT -----------------------------------

$PROBLEM TACROLIMUS BODYCOMPOSITION

$INPUT ID TIME DTIM AMT DV MDV EVID II ADDL SS FLAG CREA GFR HCT ALB AGE SEKS HGT WGT BMI OHYD LTM LTI LTID ATM FTI FTID FH HKK BSA IBW LBW FAT NEXP DROP AVIER

$DATA DATA.csv

$ABBREVIATED DERIV2=NOCOMMON

----------------------------------- PHARMACOKINETIC MODEL -----------------------------------

$SUBROUTINE ADVAN4 TRANS4

$PK

____COVARIATES V2____

V2COV=1

____COVARIATES CL____

IF(AVIER.EQ.0) CLAVIER = 1

IF(AVIER.EQ.1) CLAVIER = THETA(14)

IF(AVIER.EQ.999) CLAVIER = 1

IF(NEXP.EQ.0) CLNEXP = 1

IF(NEXP.EQ.999) CLNEXP = 1

IF(NEXP.EQ.1) CLNEXP = THETA(13)

CLHCT = ((HCT/0.34)**THETA(12))

CLCREA = ((CREA/135)**THETA(11))

CLALB = (ALB/42)**THETA(10)

CLAGE = ((AGE/56)**THETA(9))

CLFH= (FH/4.8)**THETA(15)

____COVARIATES CL COMBINED ____

CLCOV = CLAGE*CLALB*CLCREA*CLHCT*CLNEXP*CLAVIER*CLFH

FLAG1=0

FLAG2=0

FLAG3=0

FLAG4=0

FLAG5=0

FLAG6=0

FLAG7=0

FLAG8=0

FLAG9=0

FLAG10=0

IF(FLAG.EQ.1) FLAG1=1

IF(FLAG.EQ.2) FLAG2=1

IF(FLAG.EQ.3) FLAG3=1

IF(FLAG.EQ.4) FLAG4=1

IF(FLAG.EQ.5) FLAG5=1

IF(FLAG.EQ.6) FLAG6=1

IF(FLAG.EQ.7) FLAG7=1

IF(FLAG.EQ.8) FLAG8=1

IF(FLAG.EQ.9) FLAG9=1

IF(FLAG.EQ.10) FLAG10=1

ALAG1 = THETA(8)

KA = THETA(3)

TV2 = THETA(4)

TVV2 = V2COV*TV2

V2 = TVV2 * EXP(ETA(2))

TVV3 = THETA(6)

V3 = TVV3 * EXP(ETA(3))

TVQ = THETA(7)

Q = TVQ * EXP(ETA(4))

TVCL = THETA(5)*CLCOV

ECLA=FLAG1*ETA(5)+FLAG2*ETA(6)+FLAG3*ETA(7)+FLAG4*ETA(8)+FLAG5*ETA(9)+FLAG6*ETA(10)+FLAG7*ETA(11)+FLAG8*ETA(12)+FLAG9*ETA(13)+FLAG10*ETA(14)

CL = TVCL * EXP(ETA(1)+ECLA)

K = CL / V2

K23 = Q / V2

K32 = Q / V3

S2 = V2 / 1000

$ERROR

IPRED=F

Y=F + F*EPS(1)*THETA(1)

IF(DV.EQ.0) IPRED=0

W=SQRT(F*F*THETA(1)**2)

IRES=DV-IPRED

IWRES=IRES/W

$THETA

(0,0.245) ; 1

(0 FIX) ; 2

(3.58 FIX) ; 3

(692 FIX) ; 4

(23) ; 5

(5340 FIX) ; 6

(11.6 FIX) ; 7

(0.38 FIX) ; 8

(-0.43 FIX) ; 9

(0.43 FIX) ;10

(-0.14 FIX) ;11

(-0.76 FIX) ;12

(1.63 FIX) ;13

(0.8 FIX) ;14

(-1,0.01,10) ;15

$OMEGA

0.149 ; 1

0.242 FIX ; 2

0.281 FIX ; 3

0.619 FIX ; 4

$OMEGA BLOCK(1)

0.0185

$OMEGA BLOCK(1) SAME

$OMEGA BLOCK(1) SAME

$OMEGA BLOCK(1) SAME

$OMEGA BLOCK(1) SAME

$OMEGA BLOCK(1) SAME

$OMEGA BLOCK(1) SAME

$OMEGA BLOCK(1) SAME

$OMEGA BLOCK(1) SAME

$OMEGA BLOCK(1) SAME

$SIGMA 1 FIX

$ESTIMATION SIG=3 MAXEVAL=9999 NOABORT POSTHOC PRINT=5 METHOD=1 INTERACTION

$COVARIANCE PRINT=E UNCONDITIONAL
